# Use of point-of-care testing and early assessment model reduces length of stay for ambulatory patients in an emergency department

**DOI:** 10.1186/s13049-016-0319-z

**Published:** 2016-10-18

**Authors:** Meri Kankaanpää, Maria Raitakari, Leila Muukkonen, Siv Gustafsson, Merja Heitto, Ari Palomäki, Kimmo Suojanen, Veli-Pekka Harjola

**Affiliations:** 1 University of Helsinki, Helsinki, Finland; 2 Department of Emergency Medicine and Services, Helsinki University Hospital, Helsinki, Finland; 3 Department of Clinical Chemistry and Haematology, HUSLAB, Helsinki, Finland; 4 University of Tampere, Kanta-Häme Central Hospital, Hämeenlinna, Finland

**Keywords:** Point-of-care testing, POCT, Early assessment team, EAT, Emergency department, ED, Length of stay, LOS

## Abstract

**Background:**

To assess whether the use of point-of-care testing (POCT) and early assessment team (EAT) model shortens emergency department (ED) length of stay (LOS).

**Methods:**

This prospective, observational study with comparison between three study periods was performed in three phases in a metropolitan ED with 57,000 annual visits. Data were collected from adult ambulatory patients who were discharged home. Phase 1 served as a control (*n* = 1559 in one month). In phase 2, a comprehensive POCT panel including complete blood count, sodium, potassium, glucose, C-reactive protein, creatinine, alkaline phosphatase, alanine aminotransferase, bilirubin, amylase, and D-dimer was launched (*n* = 1442 in one month). In phase 3 (*n* = 3356 in subsequent two months), POCT approach continued. In addition, the working process was changed by establishing an EAT consisting of an emergency medicine resident and a nurse. The team operated from 12 noon to 10 p.m. was. The primary outcome was LOS (hh:mm) in the ED. Waiting times for patients requiring laboratory testing were analysed also, including time from admission to laboratory blood sampling (A2S interval), time from blood sampling to results ready (S2R interval) and time from results to discharge (R2D interval).

**Results:**

Median LOS of patients requiring laboratory tests in phase 1 was 3:51 (95 % confidence interval 03:38–04:04). During phase 2, introduction of POCT reduced median LOS by 29 min to 03:22 (03:12–03:31, *p* = 0.000). In phase 3, the EAT model reduced median LOS further by 17 min to 03:05 (02:59–03:12, *p* = 0.033). Altogether, the process was expedited by 46 min compared with the phase 1. Surprisingly, A2S interval was unaffected by the interventions among all patients needing laboratory testing. In comparison to phase 1, shortening of S2R interval was observed in phase 2 and 3, and that of R2D interval in all patients with laboratory assessments in phase 3.

**Discussion:**

The present study included adult ambulatory patients and is the first one to examine the impact of comprehensive POC test panel, first alone and then with additional process change. As a result, LOS was reduced significantly for patients needing laboratory tests. Considerable shortening in LOS came from introduction of POCT, and EAT process decreased the LOS further. We used a comprehensive POC test panel in order to maximise the patient population benefiting from the positive impacts of POC on laboratory turnaround time and length of stay. In EAT, diverse setups exist, and these differences affect the interpretation of results. The process changes in phase 3 were done by rearranging work shifts and no extra resources were added. Regarding to staffing the process improvement was thus cost neutral.

**Conclusions:**

The advantage of POCT alone compared with central laboratory seemed to lie in shorter waiting times for results and earlier discharge home. Moreover, POCT and EAT model shorten LOS additively compared with conventional processes. However, a longer time is seemingly needed to adopt a new working process in the ED, and to establish its full benefit.

## Background

Crowding of emergency departments is a universal phenomenon. Streamlining the patient flow reduces crowding, and LOS is a critical factor in flow improvement.

The current literature on POCT suggests that LOS can be reduced [1, 2]. However, several studies have focused only on specific tests and corresponding patient groups, especially chest pain patients [3–7]. Eventually, the spectrum of test panel seems to define how big of an impact POCT can potentially have on length of stay at the ED level [8]. LOS as a whole is affected by several factors. Moreover, it should be kept in mind that reduced LOS of one subgroup doesn’t necessarily affect other groups or reduce general ED crowding [9]. Contrary results have also been reported, in which POCT alone did not improve LOS [10], or had effect only on a limited subgroup of patients [11].

In order to get the full benefit out of POC testing, working models should support the process. Changes in the working model impact the whole ED patient population, not just the ones needing laboratory tests. Several studies have investigated the effect of different triage models on ED patient LOS and early assessment model (EAT) is one modification of these. The main idea has been to have a senior consultant and a nurse perform triage process. This has had positive effects on LOS or waiting times, although increased cost level has been seen as a problem in some of the studies [12–14]. As a whole, only limited research on POCT and EAT together has been done. A recent study combining these two reported a significantly shortened LOS [15].

Since studies have usually had a restricted test panel, a limited patient group or POCT implemented without any development of processes, this present study is targeted to cover all of these. The aim of this study is to compare ED length of stay, first after implementation of POCT for a comprehensive test panel, and second after establishment of an EAT. Our hypothesis is that both interventions additively reduce ED length of stay compared with the traditional process using central laboratory services.

## Methods

### Study design

The study was performed as a prospective, observational study with comparison between three study periods in a Finnish metropolitan hospital. There are approximately 57,000 annual visits at the hospital’s ED. The patient population consists of specialty care adult patients, and outside office hours also primary care patients. Overall admission rate from the ED to hospital wards is 35 %, and the remaining 65 % of patients are discharged home. The central laboratory is located on the same site, but is operated separately, with ED being one of their clients. The central laboratory charges a fee for blood sampling and every analysis, according to a specific price list.

Nurse-led triage is performed at the reception of the ED. After registration at triage, ambulatory patients wait for an ED resident’s call at an assigned lobby area. Before changes in the process described in this study, an ED resident evaluated the patient and, if necessary, ordered laboratory and radiologic tests. Patients who required a blood test were sent back to the lobby to wait for sampling and completion of test results. The same ED resident evaluated the results and made decisions about treatment and discharge.

This study focuses on ambulatory patients needing doctor’s appointment, who are eventually discharged home (33 % of all ED patients). Patients with a life-threatening condition (only a few among ambulatory patients) were excluded. Also patients admitted to hospital were excluded from the study in order to limit the bias on length of stay deriving from complicated patient care pathways and patient bed availability on the receiving wards. Patients assessed, managed and discharged by a qualified nurse alone were also excluded.

The study consisted of three phases: Phase 1 in February 2015 was a one month control period. Phase 2 started in March 3rd when the use of POCT began, and lasted until the end of March. Phase 3 was started by establishing the EAT model in April, and the use of both the EAT and POCT continued until the end of May.

Of ambulatory ED patients who were discharged home, about 30 % needed laboratory testing. The patients were not randomized to different groups. POC tests were performed if test panel fitted the need, otherwise all needed tests were ordered from the central laboratory.

This study was approved by the department of Emergency Medicine in the Hospital District of Helsinki and Uusimaa.

### Study protocol

The central laboratory personnel validated the POCT instruments, and their diagnostic accuracy was agreed to be at a clinically acceptable level. The POCT results were controlled by using control materials and control intervals accepted by the central laboratory. In total, 32 ED nurses were trained to take blood samples from patients and do the analysis using POC devices. The POCT-trained nurses immediately analysed the blood samples with the POC devices located in the sampling room at the ED. Nurses were instructed to store samples in a fridge in order to be able to test them again in the central laboratory if any problems occurred.

Comprehensive POC test panel consisted of sodium, potassium and glucose (by Cobas b 123 POC System), CRP (by Afinion), creatinine (CREA), alkaline phosphatase (APHOS), alanine aminotransferase (ALT), total bilirubin (Bil) and amylase (AMYL) (by Reflotron Plus), D-dimer (by Cobas h232 POC system), and finally, complete blood count (by PocH-100i). All devices were supplied by Roche Diagnostics.

The POCT results were sent electronically to the central laboratory database and reported through the electronic medical record system. Data were collected from hospital, laboratory and imaging databases. The search was limited to patients who visited ED during the length of the project and whose laboratory and radiology tests were ordered by the ED in question.

In phase 3, the early assessment team worked from 12:00 noon to 10:00 p.m. The time period selected represents the ED’s busiest hours and was therefore deemed most likely to be impacted by an EAT. The EAT consisted of a nurse and an ED resident. They were instructed to examine all patients waiting in the lobby. This initial assessment consisted of nursing history, observations, compilation of an investigation plan, and execution of that plan, provided it was meaningful considering the need for laboratory tests. The EAT ordered the laboratory tests, and the tests were performed by POC devices whenever the need fitted the test menu. Other laboratory tests were ordered from the central laboratory. After getting the results, a second ED resident discharged the patient. No extra personnel resources were used.

### Outcome measures

The primary outcome was length of stay (LOS) in the ED, which was defined as the time interval between registration and discharge. Additional outcomes were waiting times for patients requiring conventional laboratory testing, including time from admission to laboratory blood sampling (A2S interval), time from blood sampling to results ready (S2R interval), and time from results to discharge (R2D interval).

### Data analysis

**Fig. 1:**
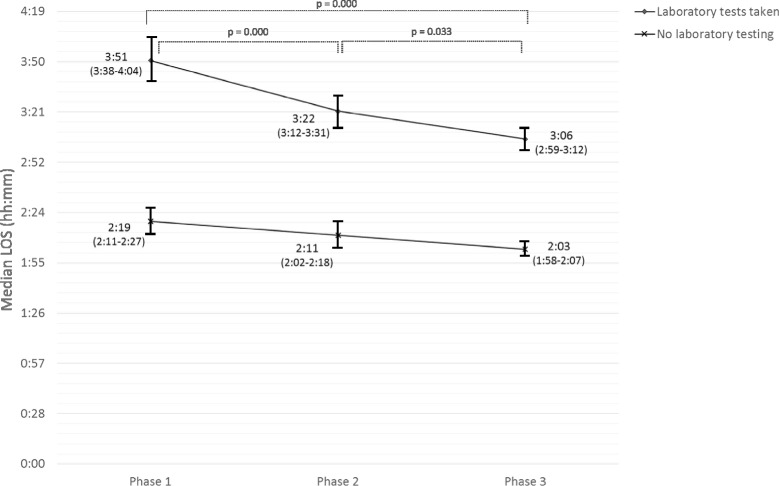
Median LOS of patient subgroups during different phases of the study [Median LOS (95 % Confidence Interval)]

**Fig. 2:**
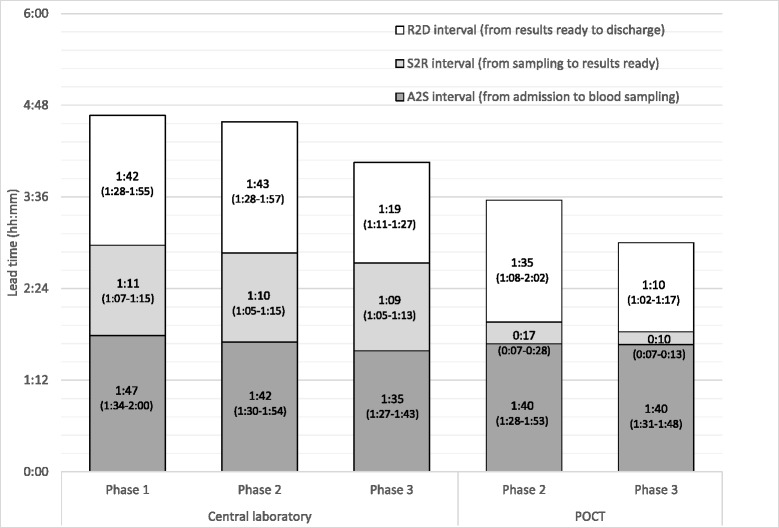
Patient waiting times from admission to sampling, from sampling to results ready and from results ready to patient discharge in central laboratory and POC groups [Mean Lead Time (95 % Confidence Interval)]

Median LOS and 95 % confidence intervals (95 % CI) were calculated and presented in Fig. 1. Mean LOS and 95 % confidence intervals (95 % CI) were used to present time intervals in detail (Fig. 2).

Mann & Whitney *U*-test was used to test for differences in LOS distributions between two subgroups, and results were presented as p-value. Statistical analyses were performed using SPSS computer software (SPSS Inc., Chicago, IL, USA). All interpretations are based on α = 0.05.

## Results

Patient characteristics were similar during each project phase. Age and gender patterns were also similar, mean age being 43 years and proportion of females being 57 %.

Any blood tests required outside the scope of these point of care tests were sent to the central laboratory for analysis. As can be seen from Table 1, despite comprehensive POC test panel, most of the ED patients needing laboratory tests had their analysis done in the central laboratory during the project. POCT percentage was better in phase 3, which implies that the EAT model improved the use of POCT. Later, all patients having at least one POCT test taken, are grouped together.

**Table 1:** Proportion of patients using POC and central laboratory tests in phase 1, 2 and 3

Patient group	Phase 1		Phase 2		Phase 3	
	Number of patients	% of total	Number of patients	% of total	Number of patients	% of total
No laboratory tests	1120	71 %	933	65 %	2286	68 %
Central laboratory tests only	439	29 %	352	24 %	617	18 %
POCT only	0	–	86	6 %	343	10 %
POCT and central laboratory tests	0	–	27	2 %	105	3 %
Inappropriate use of central laboratory despite availability of tests in POC panel	0	–	44	3 %	5	0.1 %
Grand Total	1559	100 %	1442	100 %	3356	100 %

Figure 1 shows the trend of median LOS during different project phases. LOS was reduced in every phase of the project.

Median LOS in initial control phase was 3:51 (95 % confidence interval 03:38–04:04). In phase 2, the introduction of POC testing reduced median LOS of patients needing laboratory tests by 29 min to 03:22 (03:12–03:31, *p* = 0.000). In phase 3, the launch of the EAT decreased median LOS of laboratory patients further by 17 min, to value 03:05 (02:59–03:12, *p* = 0.033). Altogether, the process was expedited by 46 min compared with the initial control phase (*p* = 0.000). A statistically significant reduction of 16 min in median LOS was also seen among patients not going to laboratory (*p* = 0.000).

Figure 2 presents waiting times of patients who had laboratory tests taken. A2S interval (shown dark grey) remained constant during each phase of the study. The biggest drop in lead time followed the introduction of POC testing and was due to a decrease in S2R interval (shown light grey). A second decrease in total lead time was observed in phase 3 when the EAT model was taken into use. This model’s advantage came from patients being discharged earlier (R2D interval, shown white).

## Discussion

The present study included adult ambulatory patients and is the first one to examine the impact of comprehensive POC test panel, first alone and then with additional process change. As a result, LOS was reduced by 46 min in total for patients needing laboratory tests. First, introduction of POCT decreased LOS by 29 min. This was mainly due to shortened S2R interval in POCT. Second, LOS decreased in phase 3 further by 17 min. This was mainly due to shorter R2D interval, most likely because patients did not need to wait for the same emergency medicine resident to become available. A2S interval remained constant during the different phases of the study.

When searching for ways to reduce LOS in emergency department, it seems reasonable to focus on interventions that affect the lead time most. This may be specific to different institutions, depending on how the care system is built. The hospital studied here had a relatively high fee for blood sampling and no pneumatic tube system in use. This setting makes the use of POC devices and ED nurse led sampling an attractive alternative.

Also patient group characteristics affect the comparability of the results. In one study in which POCT had no effect on the LOS in the ED, the admission rate of patients was as high as 85 % [16]. Another study reported similar results, as POCT had effect only on patients discharged home, and no significant impact on patients admitted to hospital [11]. Compared to the admission rate of 35 % in our study, it is clear that in the studies mentioned above, the availability of beds on wards had a larger effect on LOS and gave only a minor role for POCT.

POCT panel coverage has an impact on how widely it can affect the performance of ED by defining how comprehensively the patients can be tested by POCT. With a limited test panel, the overall impact can be limited if many patients need central laboratory analyses to complement POC [8]. For example, in a study by Parvin et al., only 4 % of patients needing laboratory tests were analysed by using POC tests only [10]. By contrast, in our study, about 30 % of patients needing laboratory testing were managed by POCT only (see Table 1). Also, the lead time of central laboratory analyses was relatively short in the study of Parvin et al. as they e.g. used pneumatic tube system for sample transport.

We used a comprehensive POC test panel of POC tests in order to maximise the patient population benefiting from the positive impacts of POC on laboratory turnaround time and length of stay. Results from studies reporting improved LOS for only limited patient groups of specific symptoms or diagnoses [3, 5–7] are limited in their application to general ED population.

In order to get full benefit out from POCT, also the EAT model was tested. This has had positive effects on LOS or waiting times, although increased cost level has been seen as a problem in some of the studies [12–14]. However, the process changes in our study were done by rearranging work shifts and no extra resources were added. Regarding to staffing the process improvement was thus cost neutral. In the long term such shortening in LOS might enable some reduction in staffing, especially after the patient inflow to ED slows down.

Again, the performance of this model depends on the system and also the individuals working within it. Many of the early assessment models presented in the literature replaced triage with an early assessment team [12, 14, 15, 17] that included an ED resident and a nurse. Thus, all the studies mentioned above differ from our setup, since in our early assessment model the triage made by a nurse at the registration point was retained. This affects the comparability of numbers, since the first waiting time is over an hour in our study, compared for example with waiting times of only minutes in the study by Jarvis et al.

This setup may also have affected the first waiting time (A2S interval) and the LOS in our EAT model, since certain urgency to assess the patient does not exist. The total LOS was reduced by the use of an EAT. In our model, the use of an EAT had the greatest impact on the waiting time to the second resident, focusing mostly on patients with results ready. However, it was somewhat unexpected that it did not affect the patients’ first waiting time (A2S interval). There seems to be room for major improvement in our EAT process. In order to get full benefit from the EAT model, nurse-led initial triage should be completely replaced by an EAT, as was done in all the above-mentioned studies.

LOS can be improved by other means also, for example Terris et al. reported improved lead time when fast patients were identified early: almost 50 % of patients were discharged after initial assessment at the triage area [14]. This naturally improves the process by reducing the number of patients waiting for care.

A study by Grant et al. had a similar early assessment model to ours (i.e. registration at the triage desk, and waiting for an assessment done by an ED resident and a nurse). They reported that waiting time to first physician assessment was reduced, but not LOS [13]. They concluded that this result may have been affected by lack of resources, since the second line junior physicians would have needed consultations from the senior physician working in the busy front line, and the total number of physicians available to provide a definitive assessment was reduced.

### Limitations

In order to include most representative number of patients in phases 1 and 2, the analysis was not restricted to a time of day. Moreover, this did not affect the comparison between phase 1 and 2. However, in the last study period, due to staffing resources the EAT approach was specifically focused to the busiest hours in our ED. Outside those hours only a minority of patients arrive to the ED and the resources are accordingly more limited which precluded the use of EAT pilot in those times. This limits to a some extent the comparability of phase 3 with the other phases. However, as the staffing is balanced with demand, the differences in flow between different times of day are minimal.

This study focused only on ambulatory adult patients discharged home, which limits its application to general ED population and paediatric patients.

The study was not randomized, which may have an effect on results.

Patients were grouped according to the need for laboratory tests. Some patients naturally needed radiology tests too. Since the number of patients was high enough and the need for radiology is quite constant, these patients were not separately analysed. However, it should be noted that these patients increase the calculated mean LOS.

## Conclusions

According to our results, POCT alone shortened the patient LOS by leaving the time-consuming central laboratory out. Although the central laboratory was still used with many patients, POCT reduced the LOS of the whole patient group examined. Thus, the use of POCT can be considered to have a notable effect on the emergency department’s performance.

Early assessment model streamlined the process and was able to shorten LOS even further. The main reason for this seems to be the better ability of the second physician to focus on the patients with results ready. However, a longer time is seemingly needed to adopt a new working process in the ED, and to establish its full benefit.

Based on these results, our ED continues using both systems. The aim is to decrease the time from admission to blood sampling by using team triage model at the registration point, instead of performing triage and EAT separately. Further analysis will be carried out on the cost-effectiveness of the POCT.

## Abbreviations

EAT: Early assessment team
ED: Emergency department
LOS: Length of stay
POCT: Point-of-care testing
